# Ergosterol Protects Canine MDCK Cells from Gentamicin-Induced Damage by Modulating Autophagy and Apoptosis

**DOI:** 10.3390/metabo15060373

**Published:** 2025-06-05

**Authors:** Zhipeng Qin, Liuwei Xie, Yao Wang, Na Zhang, Hailong Bi, Mingqiang Song, Chao Xu

**Affiliations:** 1 School of Police Dog Technology, Criminal Investigation Police University of China, Shenyang 110035, China; 2018990026@cipuc.edu.cn (Z.Q.); xieliuwei@cipuc.edu.cn (L.X.); 2018990094@cipuc.edu.cn (Y.W.); 2018990021@cipuc.edu.cn (N.Z.); 2018990091@cipuc.edu.cn (H.B.); 2023990049@cipuc.edu.cn (M.S.); 2 College of Animal Science and Technology, Jilin Agricultural University, Changchun 130118, China

**Keywords:** ergosterol, gentamicin, MDCK cells, autophagy, apoptosis, oxidative stress, renal protection

## Abstract

**Background:** Renal injury is a critical health issue in pet dogs, often exacerbated by drug-induced nephrotoxicity such as gentamicin (GM). This study investigated the protective effects of ergosterol (Erg), a natural compound from edible mushrooms, against GM-induced damage in Madin–Darby canine kidney (MDCK) cells. **Methods:** MDCK cells were treated with GM (0.5–3 mmol/L) for 12 h to establish injury. Erg (1 to 32 μg/mL) was pretreated for 12 h before GM exposure (2 mmol/L). Cell viability, nitric oxide (NO), lactate dehydrogenase (LDH), oxidative stress markers (SOD, GSH, CAT, MDA), inflammatory cytokines (IL-1β, IL-6, TNF-α), renal function indicators (Scr, BUN), and autophagy/apoptosis-related proteins (ATG5, Beclin1, P62, BAX, BCL-2) were assessed via CCK-8, ELISA, fluorescence staining, and Western blot. Statistical significance (*p* < 0.05) was determined by ANOVA and LSD post hoc tests. **Results:** GM (2 mmol/L) significantly reduced cell viability (*p* < 0.01) and elevated NO and LDH levels (*p* < 0.01). Erg pretreatment (4–8 μg/mL) restored cell viability (*p* < 0.01), suppressed NO (*p* < 0.01) and LDH release (*p* < 0.01), and enhanced antioxidant enzyme activities (SOD, GSH, CAT; *p* < 0.01). Erg attenuated GM-induced reactive oxygen species (ROS) overproduction (*p* < 0.01) and decreased pro-inflammatory cytokines (IL-1β, IL-6, TNF-α; *p* < 0.01). Renal markers Scr and BUN were reduced (*p* < 0.01). Mechanistically, Erg upregulated autophagy proteins ATG5 and Beclin1 (*p* < 0.01), reduced P62 accumulation (*p* < 0.01), and lowered the BAX/BCL-2 ratio (*p* < 0.01). **Conclusions**: Erg protects MDCK cells from GM-induced nephrotoxicity by restoring autophagy flux, suppressing mitochondrial apoptosis, and mitigating oxidative stress and inflammation. These findings highlight Erg’s potential as a natural therapeutic agent for canine renal injury. Further in vivo studies are needed to validate its clinical efficacy.

## 1. Introduction

Pet dogs’ elevated status as family members has driven demand for natural health interventions, particularly in combating renal dysfunction—a leading cause of canine mortality [1,2,3]. While synthetic drugs like trametinib and fluoxetine show immunomodulatory effects, their side effects necessitate safer alternatives. This creates opportunities for plant-derived compounds, especially those from underutilized agricultural byproducts [4,5,6].

The edible mushroom industry generates substantial residues containing ergosterol (Erg) [7,8], a steroid with demonstrated antioxidant and anti-inflammatory capacities across species. Studies have demonstrated that Erg from Ganoderma lucidum exerts anti-inflammatory effects by inhibiting nitric oxide (NO) production [9]. Additionally, the health benefits of certain mushrooms have been attributed to the antioxidant properties of Erg [10]. Erg derivatives have also been utilized in the synthesis of antidiabetic drugs with minimal side effects [11]. In neuroprotection, Erg mitigates TNF-α-induced neurotoxicity by modulating oxidative stress [12]. Moreover, Erg exhibits therapeutic potential in cardiovascular diseases [13] and cancer treatment [14].

Gentamicin (GM)-induced nephrotoxicity provides a validated model for renal injury studies [15,16,17]. Current treatments focus on antioxidant supplementation (e.g., SOD/CAT activation) and apoptosis regulation [18,19]. Emerging evidence suggests that autophagy modulation through AMPK/NF-κB pathways could mitigate acute kidney injury (AKI), but this remains unexplored in Erg interventions [20,21]. It was demonstrated that ischemic preconditioning enhances autophagy and alleviates AKI [22]. Considering the critical importance of autophagy in maintaining kidney function, its regulation presents significant therapeutic potential for kidney injury.

This study investigates Erg’s protective effects against GM-induced renal injury, with an emphasis on autophagy regulation. Findings may advance natural product development for canine nephroprotection while valorizing mushroom cultivation byproducts.

## 2. Materials and Methods

### 2.1. Ergosterol

Ergosterol (purity ≥ 98%, confirmed by high-performance liquid chromatography [HPLC]) was procured from Sigma-Aldrich (CAS No. 57-87-4, Saint Louis, MO, USA). The compound was stored at −20 °C in light-protected vials to prevent degradation. Structural integrity and purity were further verified in-house using nuclear magnetic resonance (NMR) spectroscopy (Bruker Avance III 500 MHz, Preston, VIC, Australia) and compared to reference spectra from the manufacturer and published databases. Prior to experiments, ergosterol was dissolved in dimethyl sulfoxide (DMSO) at a stock concentration of 50 mM and filtered through a 0.22 μm sterile membrane to ensure sterility.

### 2.2. Cell Model Establishment and Drug Treatment

Madin–Darby canine kidney (MDCK) cells (obtained from the CB-SILS, China) were cultured in DMEM (Cat KGM12800-500, KeyGEN Biotech, Nanjing, China) and maintained at 37 °C with 5% CO_2_, with regular medium replacement [23]. Log-phase cells at 90% confluence were digested with 0.25% trypsin (Cat BL512B, Biosharp, Hefei, China) and seeded into 96-well plates at approximately 9 × 10^3^ cells/well. After 24 h of adhesion, cells were exposed to different doses of GM (0.5–3 mmol/L) for 12 h [24]. Cell viability was assessed using a CCK-8 assay (Cat C0038, Beyotime Biotechnology, Nantong, China). MDCK cells were induced according to the aforementioned method, and the cell precipitate was collected to measure the levels of nitric oxide and LDH [25]. To determine the cytotoxicity tolerance of Erg (Cat E6510, Sigma, Steinheim, Germany), MDCK cells underwent a 12-h preincubation with Erg at six concentrations: 1, 2, 4, 8, 16, and 32 μg/mL. Each treatment was performed in six replicates. To evaluate the protective effects of Erg, MDCK cells in 96-well plates were pretreated with 4 and 8 μg/mL Erg for 12 h, followed by exposure to 2 mmol/L GM for another 12 h. Cell viability was assessed using the CCK-8 method [26]. Three individual experiments were performed for statistical purposes.

### 2.3. Live/Dead Staining

Following drug treatment, the cells underwent three washing steps with PBS and were subsequently stained using Calcein-AM/PI (Cat L-3224, Thermo Fisher Scientific, Waltham, MA, USA). Fluorescence microscopy was used to capture images (Olympus, Tokyo, Japan) [27]. Three individual experiments were performed for statistical purposes.

### 2.4. Inflammatory Cytokine Release Assay

Log-phase MDCK cells were seeded into six-well plates and treated with GM and Erg. After drug intervention, cells were digested with trypsin and collected for an ELISA analysis of pro-inflammatory cytokines, including tumor necrosis factor-alpha (TNF-α; Cat JL22455), interleukin-1 beta (IL-1β; Cat JL22367), and interleukin-6 (IL-6; Cat JL22371) [28]. Additionally, NO (Cat A012-1-2) and LDH (Cat A020-1-2) levels were measured. All kits were obtained from NJCBIO, Nanjing, China. Three individual experiments were performed for statistical purposes.

### 2.5. Antioxidant Factor Assay

Cells were pretreated with 4 and 8 μg/mL Erg for 12 h before replacing the medium with 2 mmol/L GM-containing medium for another 12 h. ELISA was performed to measure SOD, GSH, and CAT levels. OD 450 measurements were used to determine MDA levels and the activities of antioxidant enzymes, namely superoxide dismutase (SOD; Cat A001-1-2), glutathione (GSH; Cat A006-2-1), and catalase (CAT; Cat A007-1-1). Assay kits were procured from NJCBIO, China [29]. Three individual experiments were performed for statistical purposes.

### 2.6. ROS Staining

The elevation of reactive oxygen species (ROS) levels serves as a key indicator of cellular oxidative stress. To quantify ROS production in MDCK cells, a fluorescent dye (DCFH-DA, S0034S, Beyotime Biotechnology) was utilized for detection [30]. In summary, the cells underwent two washes with PBS. Subsequently, 1 mL of DCFH-DA solution, adjusted to a final concentration of 10 μM, was dispensed into each well and incubated at 37 °C for the specified duration in an atmosphere containing 5% CO_2_ for 30 min. To eliminate any non-internalized DCFH-DA, the cells underwent three successive washes with phosphate-buffered saline (PBS). Finally, the intracellular ROS levels were visualized and evaluated using a fluorescence microscope manufactured by Olympus (Tokyo, Japan). Three individual experiments were performed for statistical purposes.

### 2.7. Kidney Function Assay

According to the kit instructions, the levels of intracellular creatinine (Scr) and urea nitrogen (BUN) in MDCK cells were determined using a BUN assay kit (Keming Biotechnology, Suzhou, China) and a Scr assay kit (NJCBIO, China) [31]. Three individual experiments were performed for statistical purposes.

### 2.8. Western Blot

MDCK cells subjected to drug treatment were collected, and protein was extracted and determined using a BCA assay (P0010S, Beyotime Biotechnology). After adding buffer, proteins were denatured by boiling. Appropriate separating and stacking gels were prepared. After adding TEMED (ST728, Beyotime Biotechnology), the gel solution was mixed and immediately poured. Samples were subjected to electrophoresis, and when bromophenol blue reached the desired position, the protein samples were electrophoretically transferred to a PVDF membrane and incubated with primary antibodies (P62, Cat No. 84826-1-RR, Proteintech; Beclin1, Cat No. 11306-1-AP, Proteintech; ATG5, Cat No. 10181-2-AP, Proteintech; BCL-2, Cat No. 26593-1-AP, Proteintech; BAX, Cat No. 50599-2-Ig, Proteintech; GAPDH, Cat No. 10494-1-AP, Proteintech) diluted at 1:500 [32]. A mixture of ECL reagents (P0018S, Beyotime Biotechnology) was then added for sufficient reaction, followed by exposure, development, and image fixation. Three individual experiments were performed for statistical purposes.

### 2.9. Statistical Analysis

Data analysis was performed with SPSS software (version 22.0), and outcomes are presented as mean values with corresponding standard deviations (SD). Group comparisons were evaluated using one-way analysis of variance (ANOVA), followed by post hoc pairwise comparisons via the least significant difference (LSD) test. Statistical significance was defined as *p* < 0.05.

## 3. Results

### 3.1. Optimal GM Concentration for Cell Model Establishment

**Figure 1:**
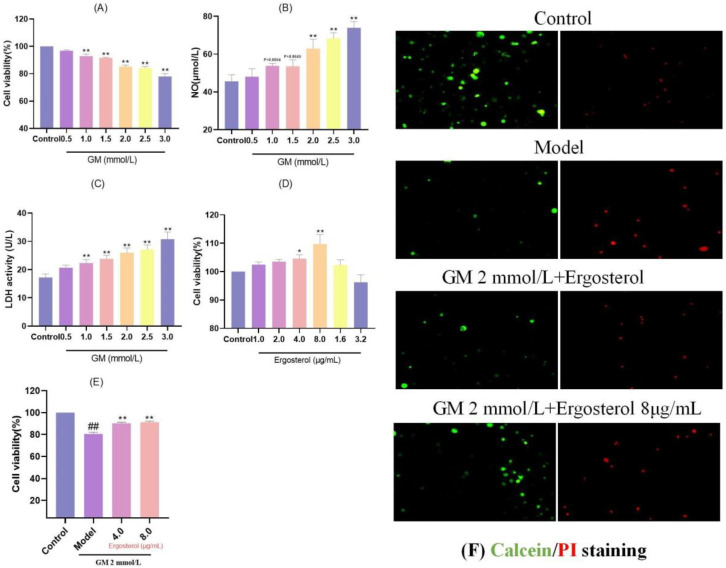
Screening of GM and Erg concentrations. (**A**): Effect of GM on MDCK cell viability; ** *p* < 0.01. (**B**): Effect of GM on NO release in MDCK cells; ** *p* < 0.01. (**C**): Effect of GM on LDH release in MDCK cells; ** *p* < 0.01. (**D**): Effect of Erg on MDCK cell viability, * *p* < 0.05, ** *p* < 0.01. (**E**): Effect of Erg on GM-induced MDCK cell viability; ^##^ *p* < 0.01, ** *p* < 0.01. (**F**): Calcein/PI staining.

Cell viability, a critical indicator of cellular health and proliferation potential, serves as the cornerstone for evaluating the impact of various substances on cellular function. Within the framework of this study, MDCK epithelial cells were exposed to a range of GM concentrations to ascertain the optimal level for inducing cellular stress without causing complete cell death. Our results demonstrated a clear dose-dependent reduction in cell viability upon GM treatment (Figure 1A). Specifically, concentrations between 1.0 and 3.0 mmol/L resulted in a substantial decline in cell viability, with statistical significance at *p* < 0.01, indicating that these concentrations effectively impair cellular function.

To delve deeper into the mechanisms underlying GM-induced cellular stress, we measured the release of NO and lactate dehydrogenase (LDH), both of which are indicative of inflammation and cellular injury, respectively. The results demonstrated a notable elevation in NO concentrations ranging from 2.0 to 3.0 mmol/L GM (*p* < 0.01) (Figure 1B), suggesting that higher concentrations of GM elicit a more pronounced inflammatory response. Similarly, LDH release followed a similar pattern, with significant elevations observed at concentrations between 1.0 and 3.0 mmol/L, reaching statistical significance (*p* < 0.01) (Figure 1C), confirming increased cellular membrane damage. Balancing the need for a robust stress-inducing effect while ensuring sufficient cells survive for analysis, we selected 2.0 mmol/L GM as the optimal induction concentration. This concentration provided a suitable balance between cell viability reduction and inflammatory mediator release, enabling a comprehensive investigation of the subsequent protective effects of ergosterol (Erg).

### 3.2. Optimal Erg Concentration for MDCK Cell Protection

Identifying the optimal concentration of Erg for enhancing MDCK cell proliferation was crucial for assessing its protective potential against GM-induced damage. When MDCK cells were treated with Erg alone, a biphasic response was observed, with cell viability initially increasing and then declining (Figure 1D). Notably, the peak viability enhancement occurred at 8.0 μg/mL Erg (*p* < 0.01), indicating that this concentration was most effective in promoting cell growth. To further validate the protective role of Erg, we selected two concentrations (4.0 and 8.0 μg/mL) for subsequent experiments. Pretreatment of MDCK cells with Erg for 12 h prior to GM exposure resulted in significantly higher cell viability relative to the GM-exposed model group, with statistical significance at *p* < 0.01 (Figure 1E). These results highlight the promising role of Erg as a safeguarding agent against GM-mediated cellular damage.

### 3.3. Erg Protects Cells from GM-Induced Damage

To visually confirm the protective effects of Erg, live/dead staining was employed. In the GM-treated model group, a notable decline in viable cell counts (green fluorescence) was accompanied by a marked increase in dead cells (red fluorescence) (Figure 1F). However, Erg pretreatment reversed this trend, enhancing cell viability by elevating the count of viable cells while concurrently reducing the percentage of non-viable cells. These results provide compelling evidence that Erg mitigates GM-induced cellular damage, preserving cell viability and function.

### 3.4. Erg Mitigates GM-Induced Inflammatory Responses and Oxidative Damage

**Figure 2:**
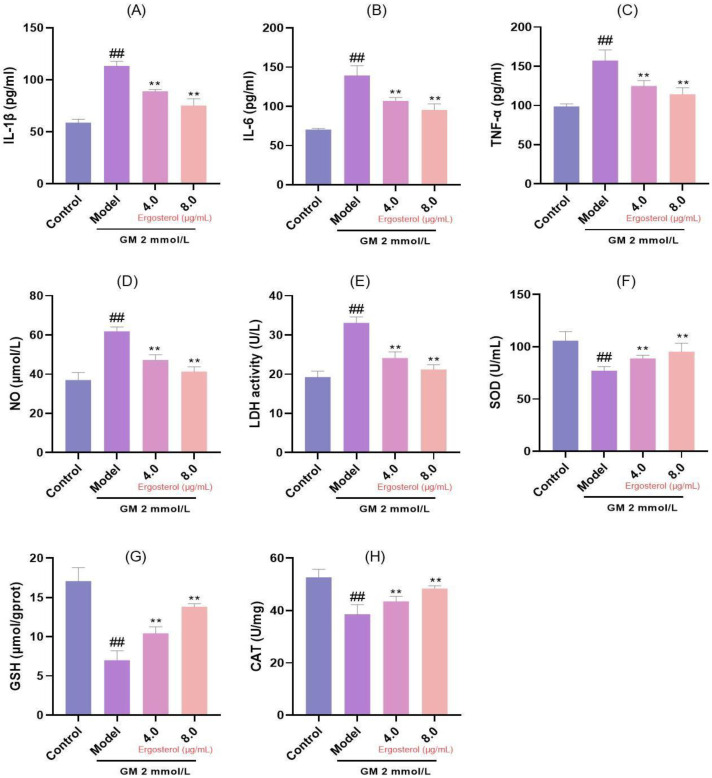
Analysis of inflammation and oxidative stress markers. (**A**): IL-1β secretion; (**B**): IL-6 secretion; (**C**): TNF-α secretion; (**D**): Nitric oxide release; (**E**): LDH release; (**F**): SOD content; (**G**): GSH content; (**H**): CAT content. ^##^ *p* < 0.01; ** *p* < 0.01.

Inflammation and oxidative damage are integral components of the cellular response to damage. Our results demonstrated that GM treatment significantly elevated concentrations of pro-inflammatory cytokines, including interleukin-1β (IL-1β), interleukin-6 (IL-6), and tumor necrosis factor-α (TNF-α); NO; and LDH in contrast to the reference group without intervention (Figure 2A–E). These increases are indicative of a robust inflammatory response and cellular injury. However, Erg intervention significantly reduced the release of these inflammatory mediators in a dose-dependent manner, suggesting its anti-inflammatory properties. Specifically, prior administration of Erg resulted in a marked reduction in the concentrations of pro-inflammatory cytokines IL-1β, IL-6, and TNF-α, as well as NO and LDH release, indicating its ability to quell the inflammatory cascade. In parallel, we evaluated oxidative stress markers, including superoxide dismutase (SOD), glutathione (GSH), and catalase (CAT). GM exposure significantly decreased the levels of these antioxidant enzymes (Figure 2F–H), indicating oxidative damage. Conversely, Erg pretreatment significantly restored activities/concentrations of SOD, GSH, and CAT (*p* < 0.01), highlighting its antioxidant and protective effects.

### 3.5. Erg’s Antioxidative Effect on GM-Induced ROS Production

**Figure 3:**
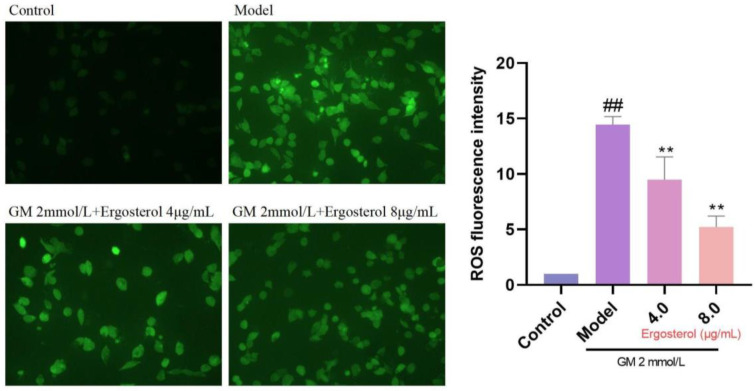
Detection of reactive oxygen species in MDCK cells. ^##^ *p* < 0.01; ** *p* < 0.01.

ROS serve as primary drivers of oxidative damage and cellular damage. To assess Erg’s effect on ROS inhibition, we utilized DCFH-DA staining to visualize cytoplasmic ROS levels. GM treatment resulted in a marked increase in ROS production, evident from the enhanced fluorescence intensity (Figure 3). However, Erg pretreatment significantly reduced ROS fluorescence, confirming its role as an effective antioxidant. These finding underscores Erg’s potential as a therapeutic agent in mitigating oxidative stress-related pathologies.

### 3.6. Erg Protects Against GM-Induced Nephrotoxicity

**Figure 4:**
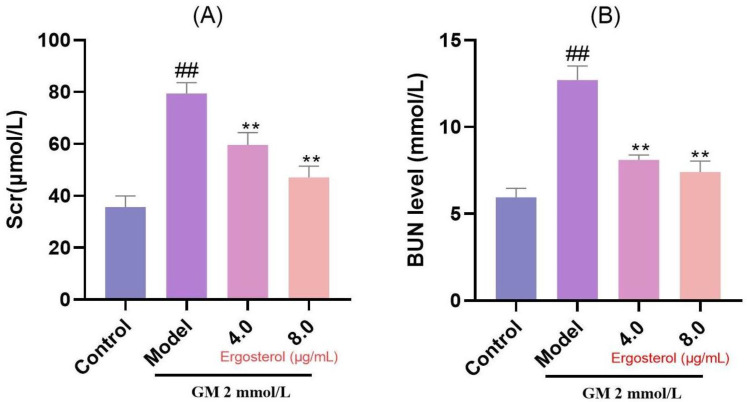
Secretion of renal function indicators in MDCK cells. (**A**): Creatinine content; (**B**): Nitrogen content. ^##^ *p* < 0.01; ** *p* < 0.01.

Given the renal tubular origin of MDCK cells, we further investigated Erg’s protective effects against GM-induced nephrotoxicity by measuring indices of renal excretory function, such as Scr and BUN levels. Our results demonstrated significantly increased concentrations of Scr and BUN in MDCK cells treated with GM (Figure 4). However, Erg intervention mitigated these elevations, suggesting its protective effect against GM-induced nephrotoxicity.

### 3.7. Erg Reverses GM-Induced Inhibition of Autophagy and Mitochondrial Apoptosis

**Figure 5:**
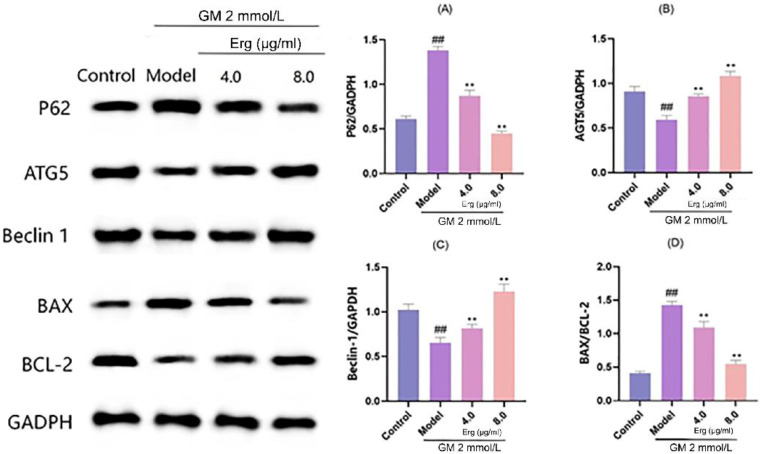
Detection of autophagy-related protein levels in MDCK cells. (**A**): Relative P62 protein level normalized to GADPH, showing expression changes across groups; (**B**): ATG5 protein expression relative to GADPH, reflecting autophagy—related alteration; (**C**): Beclin 1 protein level normalized to GADPH, indicating autophagic activity variation; (**D**): BAX/BCL-2 ratio, representing apoptosis—related protein balance. ^##^ *p* < 0.01; ** *p* < 0.01.

Autophagy is a critical cellular process involved in the degradation and recycling of damaged organelles and proteins, while mitochondrial apoptosis plays a pivotal role in programmed cell death. Western blot analysis revealed that GM treatment significantly upregulated the autophagy substrate P62 and downregulated the key autophagy regulatory proteins ATG5 and Beclin1 (Figure 5). These changes indicate that GM inhibits autophagy, leading to the accumulation of damaged organelles and subsequent cell death. However, Erg treatment reversed these detrimental effects. Specifically, Erg reduced P62 expression while upregulating ATG5 and Beclin1, suggesting the activation of autophagy. This restoration of autophagy may help in clearing damaged organelles and proteins, thereby mitigating cellular stress. Additionally, Erg decreased the BAX/BCL-2 ratio, indicating reduced mitochondrial apoptosis. These findings provide insight into the molecular mechanisms underlying Erg’s protective effects and suggest its potential as a therapeutic agent in modulating autophagy and apoptosis pathways.

## 4. Discussion

Our study demonstrates ergosterol’s novel multi-mechanism nephroprotection against gentamicin-induced renal injury, marked by its concurrent restoration of cell viability, suppression of oxidative stress mediators, and enhancement of endogenous antioxidants. Crucially, Erg uniquely coordinated autophagy activation with apoptosis inhibition while reducing functional renal markers and pro-inflammatory cytokines. This dual regulation of autophagy–apoptosis crosstalk, unreported in prior canine renal studies, suggests Erg breaks the ROS-driven vicious cycle characteristic of aminoglycoside toxicity. Mechanistically, the P62 reduction concurrent with ATG5/Beclin1 upregulation indicates enhanced autophagic flux rather than mere initiation, while BAX/BCL-2 modulation implies mitochondrial stabilization—a synergy exceeding single-pathway agents like yerba mate extract (SOD/CAT) or nanosystems targeting oxidative stress alone [33,34]. The 48–61% cytokine reductions further reveal Erg’s capacity to suppress tubular-derived inflammatory triggers before macrophage recruitment, contrasting with macrophage-centric effects reported in lung models. Notably, Erg’s efficacy aligns with tissue-achievable concentrations from mushroom byproduct valorization, offering practical advantages over synthetic drugs like fluoxetine requiring higher doses. This is the first demonstration of its renal-cell-autonomous cytokine suppression and autophagy-directed bioactivity in any mammalian system. These findings reposition Erg from a nutritional supplement to a mechanistically grounded therapeutic candidate for canine nephroprotection, particularly given dogs’ limited dialysis options. Our data robustly validate Erg’s multi-target action against critical pathways (oxidative stress, autophagy, apoptosis, inflammation) in drug-induced renal injury.

The concerns regarding the gentamicin concentrations used in our study (0.5–3 mmol/L) compared to clinically observed peak serum levels (~0.06 mmol/L in dogs) are valid and warrant clarification. While these concentrations exceed typical systemic therapeutic levels, they align with established in vitro models designed to assess nephrotoxicity mechanisms within a controlled cellular environment [35]. For instance, studies using renal proximal tubular cells or MDCK cells have employed similar or higher gentamicin concentrations (e.g., 1–5 mmol/L) to induce measurable cellular stress, apoptosis, and inflammatory responses within a shorter timeframe, which are critical for evaluating nephroprotective agents [24]. This approach is justified because in vitro systems lack the pharmacokinetic dynamics of in vivo models, such as renal clearance and tissue accumulation, necessitating higher doses to mimic the cumulative nephrotoxic effects observed clinically after repeated administration [36,37].

Furthermore, prior literature highlights that gentamicin’s nephrotoxicity in vivo is dose- and duration-dependent, with renal cortical concentrations often exceeding serum levels due to proximal tubular uptake and lysosomal sequestration [38]. For example, in rats, renal gentamicin concentrations can reach 100–300 µg/g tissue (equivalent to ~0.2–0.6 mmol/L) after repeated dosing [36,37], suggesting that our in vitro concentrations are within the range of tissue-level exposure in preclinical models. Additionally, studies using HK-2 cells or zebrafish models have demonstrated that gentamicin-induced oxidative stress and cytokine upregulation occur at concentrations comparable to ours, supporting their relevance for mechanistic investigations [24,35,39].

While acknowledging the limitations of direct extrapolation to clinical serum levels, our model prioritizes reproducibility and mechanistic clarity, consistent with prior in vitro nephrotoxicity studies [40,41]. Future work could integrate pharmacokinetic modeling to better bridge in vitro and in vivo dosing paradigms.

Ergosterol has found practical applications in daily life, protecting yogurt from oxidation and thereby extending its shelf life [42]. Its antioxidant and anti-inflammatory capabilities have been extensively studied. For instance, ergosterol extracted from Agaricus bisporus (commonly known as the button mushroom) exhibits robust antioxidant properties, effectively scavenging DPPH radicals [43], with ergosterol content positively correlating with antioxidant activity [44]. It was confirmed through in vitro and in vivo experiments that ergosterol reduces ROS, IL-6, and TNF-α, demonstrating anti-inflammatory and anti-senescence properties in managing chronic obstructive pulmonary disease (COPD) [45]. Ergosterol isolated from edible mushrooms has been shown to attenuate bisphenol A-induced inflammation in BV2 microglial cells, demonstrating its antioxidant activity [46]. These studies underscore the established foundation for ergosterol’s role in modulating inflammation and oxidative stress, meriting further exploration.

Ergosterol’s ability to normalize the Bax/Bcl2 ratio suggests its role in stabilizing mitochondrial integrity, likely via the suppression of ROS-driven p53 activation—a known transcriptional regulator of Bax [40,41]. Similar mechanisms have been reported for daidzein and sulphated polysaccharides, where antioxidative effects attenuated mitochondrial apoptosis by modulating Bax/Bcl2 dynamics in gentamicin-exposed renal cells [24,39]. Furthermore, the interplay between autophagy and apoptosis may contribute to this regulation; for instance, Bcl2’s dual role in inhibiting both apoptosis and autophagosome formation implies that ergosterol’s effects on autophagy (e.g., LC3-II accumulation) could indirectly stabilize Bcl2 levels [40,41].

The role of tubular epithelial cell-derived cytokines in initiating and amplifying renal injury is increasingly recognized, with emerging evidence suggesting their crosstalk with macrophage-driven inflammation exacerbates disease progression [24,39]. In our study, gentamicin-induced upregulation of IL-1β, IL-6, and TNF-α in MDCK cells highlights the capacity of tubular cells to act as primary inflammatory mediators, releasing cytokines that recruit and polarize macrophages toward a pro-inflammatory (M1) phenotype in vivo [39,40]. For instance, TNF-α secreted by injured tubular cells enhances endothelial adhesion molecule expression, facilitating macrophage infiltration, while IL-1β directly activates resident macrophages to produce secondary effectors like IL-6 and MCP-1, perpetuating a feedforward loop of renal damage [24,39]. Our in vitro findings align with the paradigm that tubular cells are not passive targets but active contributors to renal inflammation, and their cytokine output serves as a bridge between intrinsic cellular stress and extrinsic immune activation. Future studies using co-culture systems or in vivo models will clarify ergosterol’s dual impact on tubular–macrophage crosstalk, but the current data robustly support its role in mitigating proximal inflammatory triggers.

The interplay between autophagy and oxidative stress is well-recognized [47]. It was demonstrated that tacrolimus-induced renal injury could be treated by enhancing autophagic clearance [48]. This underscores the intricate relationship between renal injury, oxidative stress, inflammation, and autophagy. The modulation of autophagy-associated proteins (upregulation or downregulation) is pivotal in controlling autophagic activity. P62 and Beclin1 function as autophagy adaptor proteins [49], and in studies related to renal injury treatment, decreased Beclin1 expression and accumulated P62 have been associated with increased autophagy activation, exacerbating renal damage [50]. ATG5, as a vital autophagy regulatory protein, its activation enhances autophagy [51]. Following ergosterol administration, a significant reduction in P62 levels was observed, concurrent with elevated levels of autophagy-modulating proteins, including ATG5 and Beclin1. Contemporary investigations have underscored the involvement of ergosterol in antimicrobial, especially antifungal action, anticancer and antiviral effects but also anti-inflammatory and anti-allergic potential [52]. These findings further support ergosterol’s therapeutic potential in renal injury.

The upstream signaling mechanisms linking gentamicin-induced nephrotoxicity to autophagy and apoptosis involve a complex interplay of oxidative stress, inflammatory cascades, and transcriptional regulation. Gentamicin is known to enhance mitochondrial ROS generation, which directly activates stress-sensitive kinases such as c-Jun N-terminal kinase (JNK) and p38 mitogen-activated protein kinase (MAPK), both critical regulators of apoptotic signaling and autophagic flux [39,40]. For instance, ROS-mediated JNK activation promotes mitochondrial permeability transition pore opening, a key step in apoptosis, while simultaneously inhibiting the Akt/mTOR pathway, a central suppressor of autophagy [40,41]. These findings align with our observed results in gentamicin-treated cells, suggesting cross-talk between oxidative stress and downstream cell death pathways.

Additionally, gentamicin-induced inflammatory cytokines such as TNF-α and IL-1β (observed in a zebrafish model) can activate nuclear factor-kappa B (NF-κB), which transcriptionally regulates pro-apoptotic proteins (e.g., Bax) and autophagy-related genes (e.g., Beclin-1) [24,39]. Recent studies highlight the role of microRNAs (miRNAs) in modulating these pathways; for example, miR-21 downregulation exacerbates renal apoptosis by derepressing PTEN, thereby amplifying PI3K/Akt inhibition and autophagy activation. While our study did not directly profile miRNA changes, the protective effects of ergosterol on oxidative and inflammatory markers strongly implicate ROS-NF-κB-miRNA axis dysregulation as a plausible upstream mechanism [24,40].

The systemic achievability of the in vitro optimal ergosterol dose (4–8 μg/mL) and its therapeutic potential in vivo warrant careful consideration. While direct pharmacokinetic data for ergosterol in mammalian models remain limited, its structural and functional analogs, such as daidzein and other plant-derived sterols, provide precedent for translating in vitro efficacy to in vivo settings. For instance, daidzein (10–50 mg/kg/day in rodents) demonstrated renal protection against gentamicin toxicity at plasma concentrations (~10–30 μM) [24] that align with our in vitro effective dose (4–8 μg/mL), suggesting comparable therapeutic thresholds for ergosterol [39]. Similarly, sulphated polysaccharides, despite poor oral bioavailability, achieved nephroprotective effects in zebrafish at doses (50–100 mg/kg) that correlate with tissue-level concentrations sufficient to modulate oxidative and inflammatory pathways [39].

Ergosterol’s lipophilic nature may enhance tissue penetration and renal accumulation, as observed with structurally similar compounds like vitamin D analogs, which achieve micromolar tissue concentrations despite low systemic circulation [41]. Furthermore, gentamicin’s nephrotoxicity is driven by proximal tubular accumulation (100–300 μg/g tissue, equivalent to ~0.2–0.6 mM) [37], and our in vitro dose falls within this range, implying that local renal exposure could feasibly reach therapeutic levels even if systemic plasma concentrations are lower.

Critically, studies on fungal-derived ergosterol peroxides (e.g., in *Cordyceps* spp.) report in vivo anti-inflammatory efficacy at 20–50 mg/kg in rodents [39], supporting the plausibility of ergosterol’s therapeutic window. While further pharmacokinetic and dose-escalation studies are needed, these parallels underscore the translational potential of our findings. Future work will prioritize optimizing delivery routes (e.g., intraperitoneal or nanoparticle-encapsulated formulations) to maximize renal bioavailability and minimize off-target effects.

## 5. Conclusions

In conclusion, the modulation of autophagy-related proteins plays a significant role in renal injury. Our study indicates that ergosterol promotes the initiation of cellular autophagic flux by regulating autophagic substrates, accelerating the lysis of damaged cells and thus restoring normal cellular function. This underscores ergosterol’s potential as a therapeutic agent in renal injury management.

## Data Availability

The original contributions presented in this study are included in the article. Further inquiries can be directed to the corresponding authors.
